# Increasing Disease-Specific Knowledge in Patients with SLE Through a Structured One-Day Seminar: Results of a Randomized, Controlled Study

**DOI:** 10.3390/healthcare14091209

**Published:** 2026-04-30

**Authors:** Christoph Schäfer, Nancy Garbe, Florian Schmidt, Annika Seider, Katja Raberger, Andreas Wienke, Gernot Keyßer

**Affiliations:** 1 Department of Internal Medicine II, Rheumatology, University Hospital Halle (Saale), 06120 Halle (Saale), Germany; christoph.schaefer@uk-halle.de (C.S.); florian.schmidt@uk-halle.de (F.S.); gernot.keyszer@uk-halle.de (G.K.); 2 Department of Internal Medicine III, Cardiology, University Hospital Halle (Saale), 06120 Halle (Saale), Germany; annika.seider@uk-halle.de; 3 Clinic for Pediatrics I, University Hospital Halle (Saale), 06120 Halle (Saale), Germany; katja.raberger@uk-halle.de; 4 Institute of Medical Epidemiology, Biometrics and Informatics, Medical Faculty, Martin-Luther University Halle-Wittenberg, 06112 Halle (Saale), Germany; andreas.wienke@uk-halle.de

**Keywords:** systemic lupus erythematosus, SLE, patient education, health education, randomized controlled trial

## Abstract

**Highlights:**

**What are the main findings?**

- A one-day educational seminar improves disease-specific knowledge in patients with SLE.
- It reduces unmet informational needs but does not lead to lifestyle modifications.

**What is the implication of the main finding?**

- Brief educational formats are well suited for knowledge transfer but appear insufficient to induce behavioral change on their own.
- Additional motivational components are likely required to facilitate lasting lifestyle modifications.

**Abstract:**

Objective: Systemic lupus erythematosus (SLE) is a complex autoimmune disease, and its diagnosis can cause considerable anxiety and uncertainty for those affected. This study aimed to investigate the effect of a one-day educational seminar on disease-specific knowledge among patients with SLE. Additionally, the influence on subjective needs, the cognitive and emotional impact of the disease, and health-related lifestyle were examined. Methods: Patients were randomly assigned in a 1:1 ratio to an intervention group or a waiting list control group. Both groups attended the seminar. Disease-specific knowledge was measured using a multiple-choice questionnaire. The primary objective was the change in knowledge after the intervention. Results: Thirty-nine participants were included in the analysis. The mean score difference between the waiting list control group and the intervention group was 3.4 points out of a maximum of 20 (95% CI 1.8 to 5) immediately after the seminar and 1.6 (95% CI −0.6 to 3.5) three months later. Pooled data from both groups showed an increase in SLE-specific knowledge from 13.7 points to 17.3 points. Three months later, SLE-specific knowledge remained above the initial value at 15.4 points. However, no influence on lifestyle was observed. Conclusion: A one-day seminar can increase disease-specific knowledge and reduce unmet informational needs but does not lead to lifestyle modifications.

## 1. Introduction

Systemic lupus erythematosus (SLE) is a complex autoimmune disease with a distinct female predominance that can lead to significant morbidity and impairment of activities of daily life and is associated with increased mortality. However, due to extended therapeutic options, the prognosis of the disease has strongly improved in recent years with regard to life expectancy and quality of life. Patient empowerment has a favorable influence on adherence to therapy [1]. It is generally assumed that adherence to treatment increases when patients are involved in the therapeutic process in the sense of shared decision-making. Consequently, patient education and shared patient–physician decision-making have been included as one of four overarching principles in the European Alliance of Associations for Rheumatology (EULAR) recommendations for the management of SLE [2].

However, providing the necessary medical knowledge in SLE is demanding and time-consuming. The diagnosis of a chronic and potentially life-threatening disease is an exceptional psychological situation for the majority of patients. As a result, many patients are unable to absorb relevant medical information offered in the first more detailed informative interview [3,4]. Therefore, many SLE patients wish to have comprehensive education at a later stage by medical personnel with professional expertise [4]. However, necessary information is often not provided to the desired extent even at a later point in time [5]. Currently, there are no structured patient information programs for patients with SLE in Germany. For this reason, medical information in the form of written and electronically available materials is frequently provided by patient support organizations, in Germany, for example, by the Deutsche Rheuma-Liga e.V., with the affiliated Lupus Erythematodes Selbsthilfegemeinschaft e.V.

In Germany, several programs for structured training of patients with rheumatic diseases have been designed. These programs have been used widely in inpatient rehabilitation medicine [6]. Hitherto, modular outpatient training courses have mainly been offered for patients with rheumatoid arthritis (RA). Through these interventions, an important disease-specific increase in knowledge can be achieved in RA patients [7].

We hypothesize that a single-day educational program tailored to the informational needs of SLE patients is an effective intervention to increase disease-specific knowledge. Therefore, it was our intention to address the need for reliable information from medical professionals. We believe that a one-day seminar is more likely to be accepted and attended by SLE patients than more extensive educational programs involving multiple sessions. In order to gain insight into the effectiveness and feasibility of such a program, we designed a structured medical information offering in the form of a one-day seminar with the involvement of a patient support organization (Deutsche Rheuma-Liga e.V.).

## 2. Materials and Methods

### 2.1. Participants

Patients with SLE were informed about a voluntary one-day educational seminar on their underlying disease through the specialized outpatient clinic of the University Hospital of Halle (Saale), eight collaborating rheumatology practices, and the websites of patient support organizations (Deutsche Rheuma-Liga e.V. and Lupus Erythematodes Selbsthilfegemeinschaft e.V.). Interested individuals received detailed information materials, an informed consent form, questionnaires, and an invitation to attend a one-day weekend seminar.

Eligible participants were German-speaking adults with SLE aged 18 to 90 years. Exclusion criteria included high disease activity or severe comorbidities that would preclude participation in the seminar, as well as significant mental or cognitive impairments that could interfere with effective knowledge acquisition.

Patients diagnosed with SLE were randomized into two groups, the intervention group and the waiting list control group (referred to as the “control group” in the following text). Participants were allocated in a 1:1 ratio using external randomization lists with randomly defined blocks of four upon receipt of enrollment. Randomization was performed by an independent secretary not involved in patient recruitment, ensuring separation between sequence generation and enrollment. Participants, study personnel and statisticians were not blinded.

### 2.2. Intervention

Both groups attended an identical, structured, six-hour information session at different time points. Five modules covered the topics “Immune System and Immune Diseases”, “Medications and Drug Side Effects”, “Disease Pattern and Organ Manifestations of SLE”, “Diagnostics, Therapy and Lifestyle” and “Self-Help, Work and Occupation”. The first four modules lasted 45 min each, and the last module 25 min. The modules were presented by a rheumatologist or a physician with experience in the treatment of rheumatic diseases. The last module (“Self-Help, Work and Occupation”) was held by a social worker from a patient support organization (Deutsche Rheuma-Liga e.V.). After the first two modules, a 40 min lunch break was included with opportunities for interaction among participants. Life partners were allowed to attend the information session. Following all five modules, 20 min were scheduled for a final discussion and concluding questions. The topics covered in the seminar were based on the 2019 EULAR recommendations for the management of SLE and, in particular, the recommended basic measures for preventing SLE-related organ damage [8,9].

### 2.3. Questionnaires

Disease-specific knowledge. Disease-specific knowledge was measured using a self-designed multiple-choice test with 20 questions about disease progression, lifestyle, diagnosis, and treatment of SLE (Document S1). Each question came along with a choice between five different answer proposals, containing only one correct answer. All necessary information to answer the questions correctly was provided in the information session. A minimum of zero and a maximum of twenty points could be achieved in the test. The knowledge test was designed by a rheumatologist (G.K.) with extensive experience in developing multiple-choice tests for medical students. Before the trial began, the test questions were reviewed by two medical students (F.S., A.S.) and two lupus patients to ensure they were clear and easy to understand. Thereafter, minor verbal modifications were made to the questionnaire.

**Figure 1:**
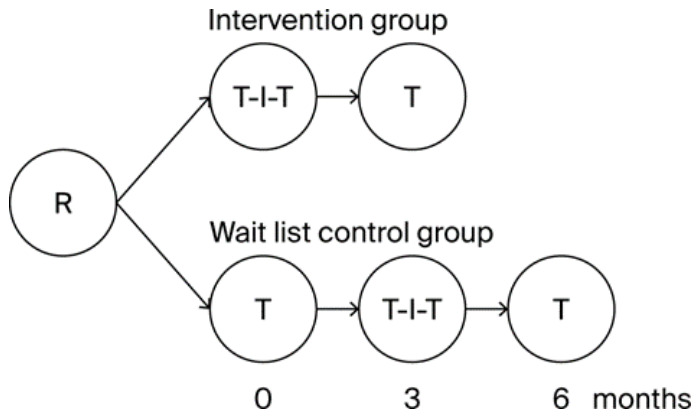
Study design. Participants were randomized (R) at baseline. Both groups received the intervention (I) consisting of a one-day seminar. Tests (T) on disease knowledge were conducted in both groups at baseline, directly after the intervention and three months after the intervention. The control group had an additional test after a three-month waiting period immediately before the intervention.

Both groups filled in the multiple-choice test at baseline after randomization. The intervention group repeated the test immediately after the intervention and three months later. The control group repeated the questionnaire after a waiting period of three months, immediately before the intervention, immediately after the intervention, and three months later (Figure 1). The control group was introduced in order to quantify or rule out any learning effect that might occur from repeated completion of identical questions. The 20 multiple-choice questions remained identical, but the order of the questions and of the answers was varied in each iteration to minimize non-specific learning effects. Participants were not informed of their performance in the evaluation or of the correct answer choices between each interview. The knowledge gain was reflected by an increase in the points achieved in the test.

SLENQ. In addition to the multiple-choice test, the survey included items assessing participants’ need for disease-specific information both prior to and three months following the intervention. This need was evaluated using the Systemic Lupus Erythematosus Needs Questionnaire (SLENQ) [10], a 97-item instrument encompassing seven domains of patient needs. The SLENQ has demonstrated good validity, satisfactory reliability, and high test–retest reliability [10].

For the purposes of this study, analysis was restricted to the 13 items pertaining to the domain of health information. Each item was rated on a five-point ordinal scale (1 = no need, 2 = need already satisfied, 3 = low need, 4 = moderate need, and 5 = high need). In addition to health information, the SLENQ addresses patient needs related to physical limitations, activities of daily living, psychological distress, social support, physician–patient relations, occupational issues, and private security.

BIPQ. To explore the cognitive and emotional representation of the SLE disease, we used a modified version of the Brief Illness Perception Questionnaire (BIPQ) [11]. The BIPQ is a nine-item scale concentrating on illness perception. The first eight items are rated using a zero to ten response scale, with higher values representing a linear increase in the dimension measured. The ninth item asks for three assumed causal factors of the disease and was skipped for this study. The test has proven validity and reliability in assessing illness perception in ill populations [11]. It has been used successfully in patients with SLE and lupus nephritis [12].

Lifestyle. Health-related behavior and medication adherence were recorded for both groups at baseline and three months after the intervention. The personal judgment of the knowledge and the importance of various health-related topics (disease-specific symptoms, treatment, adverse drug reactions, exercise, dietary recommendations, vaccinations and infection protection, osteoporosis, working life) was surveyed using questionnaires as described in [5], also with a response scale of zero to ten. Nutritional preferences were assessed using the Alternative Healthy Eating Index (AHEI 2010) [13]. Adherence to medication was evaluated using the Morisky Medication Adherence Scale (MMSA-4) [14]. The MMSA-4 consists of four questions and has a score range from zero to four. A score of zero correlates with good adherence to medication. The quantity of UV protection was measured using a four-point Likert scale, and the quality was assessed by indicating the level of protection (avoidance of open sun, long clothing, sun protection factor 30–50, sun protection factor > 50). The study participants were asked to rate themselves as smokers, ex-smokers or non-smokers. Time spent on physical activities per week was recorded in five categories (never, less than 1 h, 1 to 2 h, 2 to 4 h, >4 h).

Following the information event, study participants were asked to assess the relevance of the content and the acceptance of the educational format.

### 2.4. Primary Objective and Outcome

The primary objective was the increase in disease-specific knowledge after the educational seminar, as reflected by the results of the multiple-choice test. The primary outcome measure was the mean score difference in correct answers between the control group at baseline and after three months and the intervention group at baseline and directly after the intervention or three months after the intervention, respectively.

### 2.5. Secondary Objectives

Secondary objectives were disease-specific knowledge as measured in points scored at baseline, immediately after the intervention and three months later. Further secondary outcome measures were the mean differences between the scores at baseline, after the intervention and three months after the intervention from the pooled data of both groups; changes in the scores of the SLENQ and BIPQ; changes in nutritional preferences as measured by AHEI; changes in adherence to medication as evaluated by the MMSA-4; changes in physical activities and smoking habits; and changes in adherence to UV protection measures between baseline and three months after the intervention.

### 2.6. Statistical Analysis

We assumed that the mean number of correct responses in the questionnaire would be 10 prior to the intervention and would increase to 15 following the intervention, with a standard deviation of 2.5 in the intervention group. Based on these assumptions, a sample size of 30 participants per group was calculated to provide 95% power to detect a difference in the improvement in disease-specific knowledge.

Between-group differences were analyzed using Student’s *t*-test for normally distributed variables. Within-group comparisons before and after the intervention were performed using the Wilcoxon signed-rank test. Categorical variables were compared using the chi-square test. All statistical analyses were conducted using IBM SPSS Statistics 25 (IBM Corp., Armonk, NY, USA).

### 2.7. Research Ethics

The study was approved by the Ethics Committee of the medical faculty of the Martin Luther University Halle-Wittenberg (protocol code 2019-021; approval date: 24 May 2019). It was registered in the German Clinical Trials Register (DRKS; protocol code DRKS00016603; registration date: 10 October 2019) and conducted in accordance with the principles of the Declaration of Helsinki. Written informed consent was obtained from all participants prior to enrollment.

## 3. Results

**Figure 2:**
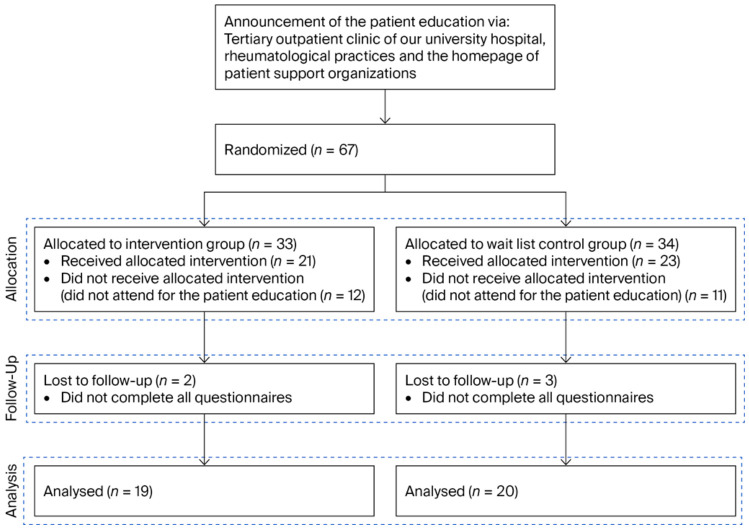
Study flow chart. Sixty-seven patients with SLE were randomized, of whom forty-four attended the seminar. Thirty-nine patients completed all questionnaires. Nineteen were assigned to the intervention group and twenty to the control group.

We randomized 67 patients with SLE, 44 of whom attended the seminar. Thirty-nine patients completed all questionnaires and were included in the further analysis (Figure 2). Nineteen were assigned to the intervention group and twenty to the control group.

The mean age of the participants was 45.5 years; SD ± 13.4 (intervention group: 46.7 years; SD ± 14.5, control group: 44.2 years; SD ± 12.5), and the mean duration of disease was 11.5 years; SD ± 9.0 (intervention group: 9.2 years; SD ± 7.4, control group: 13.5 years; SD ± 10.0). The intervention group consisted of seventeen female and two male patients, and the control group of sixteen female and four male patients. No relevant differences were found between the intervention and control groups in terms of socio-demographic and disease-specific characteristics (Table 1).

**Figure 3:**
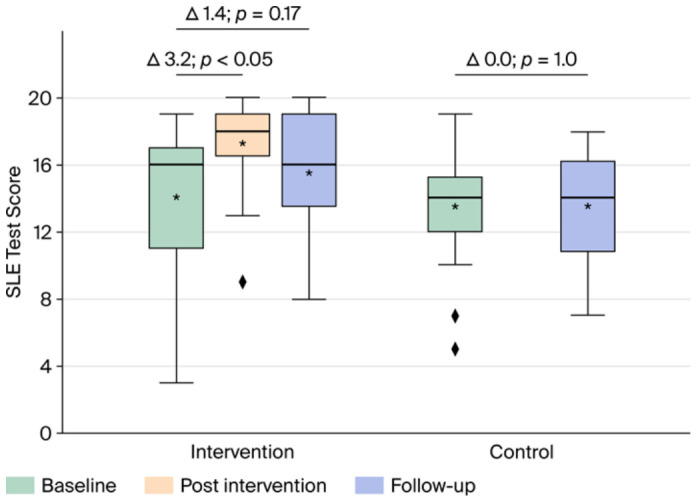
Results of the test of disease-specific knowledge of the intervention group (**left**) and the control group (**right**). Green boxes show test results at baseline in both groups. The blue box in the intervention group shows the results three months after the educational seminar. The blue box in the control group shows the results after repeating the test three months after baseline without the educational seminar. The yellow box shows the results in the intervention group immediately after the seminar. Horizontal bars indicate medians, asterisks mean values, and rhombuses outliers.

As for the primary outcome measure of the study, we found that, compared with the change in the control group, the increase in participants’ knowledge was 3.2 (95% CI 1.5 to 4.9, *p* < 0.001) immediately after the seminar and 1.4 (95% CI −0.6 to 3.5, *p* = 0.17) after three months (Figure 3).

Before the intervention, the mean number of correct answers in the intervention group was 14.1. This increased to 17.3 following the intervention and 15.5 three months after the intervention. Before the intervention, the mean number of correct answers in the control group was 13.4. Repeating the multiple-choice test after three months without intervention did not increase the score (mean score difference 0.0; SD ± 2.1).

The intervention and control groups did not differ in the mean number of correct answers at baseline. Therefore, the results of both groups were merged in the further statistical analysis of the study data.

**Figure 4:**
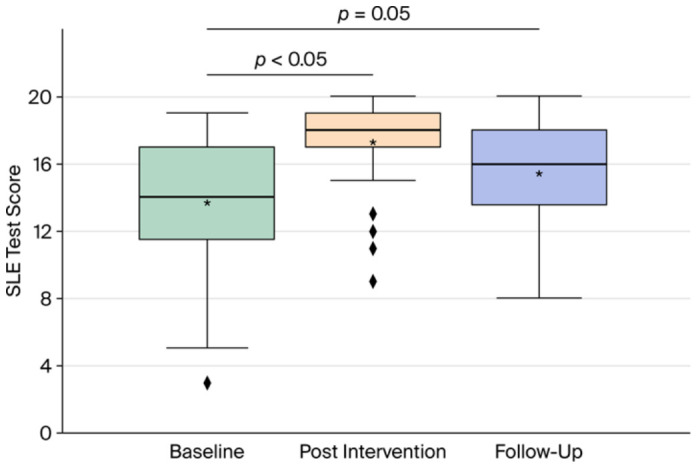
Combined test scores from the intervention and control groups (*n* = 39) at baseline, directly following the seminar, and three months after the intervention. Group means are indicated with an asterisk; group outliers are indicated with a rhombus. A minimum of 0 and a maximum of 20 points could be scored in the test.

Before the intervention, the mean score of both groups combined (*n* = 39) was 13.7 (SD ± 4.0). Immediately after the intervention, the score increased to a mean of 17.3 (SD ± 2.7), representing a mean score difference from baseline of 3.6 (95% CI 2.6 to 4.6, *p* < 0.001). Three months after the intervention, the value declined. Nevertheless, there remained a moderate increase in knowledge, with a mean of 15.4 correct answers (SD ± 3.3), representing a mean score difference of 1.7 (95% CI 0.5 to 2.7, *p* < 0.005) (Figure 4).

**Figure 5:**
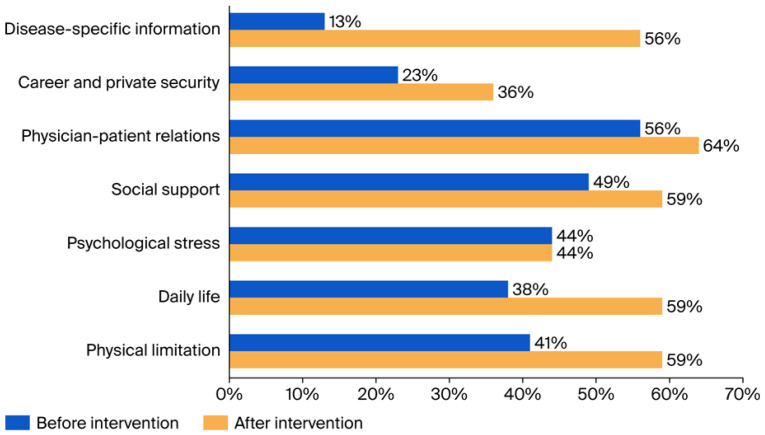
Percentage of patients with satisfied needs (“no need” or “need already satisfied”) in different domains of SLENQ before and three months after the educational one-day seminar.

Before the intervention, the majority of participants expressed unmet needs for disease-specific information, career and private security, daily life, physical limitations, psychological stress and social support (Figure 5). The most relevant need was indicated for disease-specific information. Only 13% of patients considered their disease-specific information needs to be met before the intervention. Three months after the intervention, there was a decrease in unmet needs for all topics except psychological stress. In particular, the pronounced deficit regarding disease-specific information before the event showed improvement three months after the intervention (*p* < 0.001) (Figure 5 and Figure S1).

Consistent with the results of the SLENQ, we found an increased understanding of the rheumatic disease reported in the BIPQs three months after the intervention (*p* < 0.01) (Figure S2). In parallel, there was a subjective reduction in impairment (*p* < 0.02). However, pain, fatigue, and perceived disease activity did not improve within the three months (Figure S2).

Of various disease-specific topics, “disease-specific symptoms” and “therapies” were rated as important or very important by more than 80% of the participants before the seminar (Figure S3). Three months after the seminar, there was no change in the rating of the importance of individual topics compared to the baseline data.

Osteoporosis, adverse drug effects, family planning, and professional life were the topics on which participants felt least informed before the event. Three months after the event, the number of participants who felt well informed increased for all topics (Figure S4).

Three months after the intervention, no change in participants’ health behaviors was measurable compared to the baseline data. Even before the information event, nearly all participants paid attention to sufficient UV protection by means of sunscreen and avoidance of direct sunlight. This proportion did not increase as a result of the event. Neither an increase in physical activity nor a reduction in nicotine consumption or improvement in dietary habits, as recorded by the AHEI score, was detectable in the post-intervention comparison (Table S1).

Since 92.3% of the participants already had high adherence to medication with an MMSA-4 score of zero before the intervention, no major improvement was achieved after the intervention (94.9% with high adherence post-intervention).

In response to the question “From where have you received information about your disease so far?” the treating rheumatologist ranked first for the majority of respondents, followed by internet-based sources of information. Other sources of information, in descending order of importance, are given in Figure S5. Six participants stated that they had already attended one or more disease-specific seminars on SLE.

The majority of participants evaluated the event as positive. On a five-step Likert scale (1 = complete agreement, 5 = complete disagreement), the statement “The event is helpful” received a mean of 1.51. A total of 90% of participants rated the event as helpful (complete agreement or agreement). A score of 1.68 was given in the affirmative to the question of whether there was a willingness to attend a similar event again. The question of whether the event would be recommended by participants was also answered positively by 95% of participants and was rated with a mean score of 1.31.

## 4. Discussion

There is an unmet need for reliable health information for patients with rare and complex diseases like SLE [15,16]. The aim of our study was to address this need by a one-day seminar focused on disease-specific knowledge. By improving disease-specific knowledge in patients with SLE, we intended to develop increased health literacy and treatment adherence, and thus better disease control.

Publications on structured patient education for SLE patients are scarce. Few publications focus on the influence of educational programs on the increase in knowledge of patients with SLE. A Finnish study examined the effect of reading a guide specifically designed for SLE patients and demonstrated a significant increase in disease-specific knowledge eight to ten weeks after the intervention [17]. A German study group designed and evaluated a multi-day patient educational program for SLE patients, which included five 90 min modules in small groups. The first three modules focused on knowledge about the disease; the last two modules included psychoeducational elements. Here, a significant increase in knowledge following the training program could be demonstrated in 92 patients. Knowledge increase was still detectable after six to nine months [18]. An English study group investigated the effect of a website with SLE-specific educational content on disease knowledge. After visiting the website, a significant increase in disease-specific knowledge was measured in the 42 patients examined [19]. Another study found significant improvements in coping skills following psychoeducational training for 34 patients with SLE. However, disease-specific knowledge was not evaluated [20]. An English pilot study evaluated the effects of collective education on knowledge, mood and behavior change in 12 SLE patients. In this study, possible effects of the training were recorded in patient interviews and not measured parametrically [21]. A randomized study in 80 patients with SLE showed significant improvements in fatigue and self-efficacy as an effect of a web-based education program. Again, disease-specific knowledge was not evaluated [22].

In the past, the permanent implementation of outpatient training programs for patients with SLE failed in Germany. This was mainly due to unclear funding [6,23]. The situation is better in the context of inpatient rehabilitation, where structured patient training is part of the program financed by health and pension funds [6].

Data also exist on knowledge transfer for other rheumatic diseases. In Germany, an outpatient training program was designed for RA in collaboration with rheumatology societies and patient support organizations. Following an educational program consisting of three 90 min modules, a significant increase in knowledge was documented by means of multiple-choice questionnaires [7].

In contrast to the above-mentioned educational programs for patients with SLE or RA, our educational seminar was limited to one day. With up to 23 participants, our groups were larger than in the previous publications. At the same time as the study presented in this paper, an educational seminar for patients with granulomatosis with polyangiitis was conducted by our research group, the results of which have already been published [24]. This parallel study on another rheumatic disease showed a significant and sustained increase in knowledge as a result of a one-day educational seminar. The organization of an educational seminar for larger groups of patients at a specialized center on a single day could represent an alternative that is easier to organize and finance than programs with multiple modules on different days. In our study, we focused on the gain in knowledge without the inclusion of specific psychoeducational interventions (e.g., biofeedback, relaxation, exercise, cognitive techniques to manage pain, or strategies to effect behavioral change).

This study shows that a disease-specific one-day educational program for SLE patients can achieve a measurable increase in disease-specific knowledge, indicating that this educational format is capable of strengthening the health literacy of affected patients. This contributes to the prerequisites for patients to participate as informed partners in medical decision-making.

However, the study also found that the increase in disease-specific knowledge evident immediately following the seminar weakened over the course of three months. Three months after the intervention, patients still felt significantly better informed about their disease according to the results of the BIQP questionnaire (Figure S2) and showed less unmet need for disease-specific information in the SLENQ (Figure 5). Nevertheless, at this point, a significant increase in disease-specific knowledge could only be demonstrated after combining the data from the control and intervention groups (Figure 4). Possibly, low-threshold and, e.g., web-based information services [19,22,25] could maintain the acquired disease knowledge over a longer period of time. As the majority of participants showed a willingness to participate in a similar seminar again, it would also be conceivable to repeat it at extended intervals.

The pre-existing unmet need for health information as assessed by the SLENQ was satisfied by the program for a majority of participants. The need for help and support decreased, and this change was most pronounced in the domain of “disease-specific information” (Figure 5). This improvement was well above the range observed in a longitudinal study of SLE patients without intervention over a six-month period [26].

It is likely that patients willing to attend an informational session about their disease have a greater than average need for information, indicating a possible source of sampling bias. On the other hand, our findings are consistent with other studies on SLE patients. Even in studies not offering knowledge transfer, the greatest unmet need was improved education about the disease and its treatment [25,27,28]. Although the focus on knowledge transfer also had a positive impact on other domains, especially daily life, the percentage of participants with unmet needs in the domain of psychological stress did not decrease (Figure 5).

Several limitations must be taken into account when interpreting our finding. A key limitation was that the intended sample size of 30 participants per group could not be achieved. This underpowering likely explains the lack of sustained statistical significance at three months. The fact that the combined results for the intervention group and the control group (*n* = 39) show a significant increase in knowledge after three months suggests that the sample size was insufficient to demonstrate this effect in a randomized controlled trial design.

A further major limitation was that the disease-specific knowledge test was self-designed and not validated. To date, there are no validated disease-specific knowledge tests for patients with SLE. An inadequate validation may also have contributed to the higher-than-expected number of correct answers prior to the start of the intervention. Therefore, the baseline knowledge scores were relatively high, thus confining the amplitude of measurable improvement. This ceiling effect significantly limited the ability to measure increases in knowledge and, consequently, the statistical power of the study. Future studies could consider more nuanced or advanced knowledge assessments.

Generalizability of our results may be limited due to sampling bias, as participation was voluntary. It can be assumed that participants represent a more motivated and health-literate subgroup of SLE patients. In addition, study participation criteria excluded SLE patients unlikely to comprehend or fully attend the program due to age, language barrier or disease activity. This limitation on generalizability cannot be avoided given the nature of the intervention.

A sampling bias may have influenced the results regarding behavioral changes. Significantly, our educational seminar did not result in behavioral changes three months after the intervention. The greatest changes were expected in areas of behavior that could be improved by the participants with relatively little effort. These were adherence to medication and the application of UV protection. However, the adherence to treatment reported in the MMSA-4 was already unusually high before the event (92.3%) and could hardly be improved. This percentage is considerably higher than the 37.9% [29] and 31.7% [30] previously reported for other SLE cohorts. This may be partly due to the fact that our cohort of voluntary participants had a particularly high motivation for medication adherence. The same applied to the UV protection recommended for SLE patients, which 97% of patients were already practicing prior to the intervention. There was no further room for improvement in either domain.

There is evidence that an increase in knowledge alone may not be sufficient to lead to behavioral changes. Rather, there is often a so-called “knowledge–behavior gap,” in which knowledge is not translated into corresponding action [31,32]. Lifestyle modifications with regard to smoking, exercise and diet are difficult to achieve even with more intensive interventions [33,34]. It is not surprising that a one-day educational event does not lead to a lasting change in eating, nicotine consumption habits or physical activity. Since this study focused primarily on knowledge acquisition, motivation and emotion were not measured using separate psychometric instruments. Additional motivational strategies are likely to be necessary to bring about lasting behavioral changes.

A further methodological consideration relates to allocation concealment. While randomization was conducted using externally generated allocation lists by a secretary not involved in patient recruitment, formal allocation concealment procedures (e.g., central randomization or sequentially numbered, sealed opaque envelopes) were not implemented. Nevertheless, the separation between sequence generation and enrollment, together with consecutive patient recruitment, reduces the likelihood of selection bias. In addition, baseline characteristics were well balanced between the groups, suggesting that any potential impact on group comparability is minimal.

The patients’ medical information was collected via questionnaires and could not be validated independently. Information on laboratory values and autoantibodies was not collected. It was therefore not possible to determine whether patients suffered from organ involvement and had experience with highly active SLE flares. However, the self-report of kidney involvement in about a quarter and joint involvement in over half of the participants (Table 1) suggests that our patients represented a sample with relevant systemic manifestations.

## 5. Conclusions

Educating patients with SLE in a one-day seminar can increase disease-specific knowledge without sustainable changes in health behavior. This training can be realized at a medical center with a moderate time investment for patients and medical professionals. However, it appears that additional motivational factors are needed to bring about behavioral changes.

## Figures and Tables

**Table 1:** Baseline characteristics of participants. Values are the number (%) of patients. APS, anti-phospholipid syndrome; AZA, azathioprine; BMI, body mass index; CsA, ciclosporin; HCQ, hydroxychloroquine; MMF, mycophenolate mofetil; MTX, methotrexate; NSAID, non-steroidal anti-inflammatory drug; SD, standard deviation.

Characteristics	Total (*n* = 39)	Intervention (*n* = 19)	Control (*n* = 20)
Age, y (mean ± SD)	45.5 ± 13.4	46.8 ± 14.5	44.2 ± 12.5
Gender, *n* (%)			
Men	6 (15.4)	2 (10.5)	4 (20.0)
Women	33 (84.6)	17 (89.5)	16 (80.0)
BMI (kg/m^2^), (mean ± SD)	24.7 ± 4.4	24.8 ± 5.6	24.7 ± 3.1
Education, *n* (%)			
Less than high school	20 (51.3)	9 (47.4)	11 (55.0)
High school	19 (48.7)	10 (52.6)	9 (45.0)
Employment, *n* (%)			
Employed/self-employed	19 (48.8)	9 (47.4)	10 (50.0)
Unemployed	4 (10.3)	3 (15.8)	1 (5.0)
Retired	16 (41.0)	7 (36.8)	9 (45.0)
Smoker, *n* (%)			
Current smoker	8 (20.5)	3 (15.8)	5 (25.0)
Non-current smoker	18 (46.2)	9 (47.4)	9 (45.0)
Past smoker	13 (33.3)	7 (36.8)	6 (30.0)
Duration of SLE, y (mean ± SD)	11.5 ± 9.0	9.2 ± 7.4	13.5 ± 10.0
Manifestation, *n* (%)			
Cardio-Vascular	6 (15.4)	4 (21.1)	2 (10.0)
Kidney	11 (28.2)	6 (31.6)	5 (25.0)
Joints	22 (56.4)	12 (63.2)	10 (50.0)
Blood	19 (48.7)	11 (57.9)	8 (40.0)
APS	7 (17.9)	5 (26.3)	2 (10.0)
Skin	21 (58.3)	12 (63.2)	9 (45.0)
Pulmonary system	3 (7.7)	2 (10.5)	1 (5.0)
Nervous system	1 (2.6)	0 (0)	1 (5.0)
Gastrointestinal system	1 (2.6)	0 (0)	1 (5.0)
Comorbidities, *n* (%)			
Arterial hypertension	12 (30.8)	6 (31.6)	6 (30.0)
Hypercholesterinemia	6 (15.4)	3 (15.8)	3 (15)
Arthrosis	10 (25.6)	6 (31.6)	4 (20.0)
Diabetes mellitus	0 (0)	0 (0)	0 (0)
Osteoporosis	4 (10.3)	2 (10.5)	2 (10.0)
Myocardial infarction	1 (2.6)	0 (0)	1 (5.0)
Pulmonary disease	3 (7.7)	3 (15.8)	0 (0)
Stroke	1 (2.6)	1 (5.3)	0 (0)
Malignancy	1 (2.6)	1 (5.3)	0 (0)
Current therapy regimen, *n* (%)			
HCQ	32 (82.1)	17 (89.5)	15 (75.0)
MTX	2 (5.1)	0 (0)	2 (10.0)
AZA	10 (25.6)	4 (21.1)	6 (30.0)
MMF	9 (23.1)	5 (26.3)	4 (20.0)
CsA	1 (2.6)	0 (0)	1 (5.0)
NSAID	12 (30.8)	7 (36.8)	5 (25.0)
Glucocorticoids	31 (79.5)	15 (78.9)	16 (80.0)
Belimumab	1 (2.6)	0 (0)	1 (5.0)

## Data Availability

Most of the data supporting the findings of this study are included in the article and its Supplementary Materials. Further aggregated data are available from the corresponding author upon reasonable request. Individual-level data cannot be shared due to legal and ethical restrictions related to German data protection regulations.

## Supplementary Materials

The following supporting information can be downloaded at: https://www.mdpi.com/article/10.3390/healthcare14091209/s1, Figure S1: Need for health information; Figure S2: Illness perception; Figure S3: Important topics; Figure S4: Information content; Figure S5: Sources of disease-related information; Table S1: Health-related lifestyle behavior; Document S1: Multiple-choice test.

## Abbreviations

AHEI	Alternative Healthy Eating Index
BIPQ	Brief Illness Perception Questionnaire
EULAR	European Alliance of Associations for Rheumatology,
MMSA	Morisky Medication Adherence Scale
RA	Rheumatoid Arthritis
SLE	Systemic Lupus Erythematosus
SLENQ	Systemic Lupus Erythematosus Needs Questionnaire
