# Supplementary material for: Increasing Disease-Specific Knowledge in Patients with SLE Through a Structured One-Day Seminar: Results of a Randomized, Controlled Study
Source: Healthcare (Basel). 2026 Apr 30;14(9):1209. doi: 10.3390/healthcare14091209 (PMC13163604; doi:10.3390/healthcare14091209)
Supplement: Supplementary file 1 [file healthcare-14-01209-s001.zip › Document S1.pdf]

**1. Welche Aussage zum Lupus stimmt?**	**1. Which statement about lupus is correct?**
a) Lupus kann lebenswichtige Organsysteme befallen	a) Lupus can affect vital organ systems
b) Lupus ist eine sehr häufige Erkrankung	b) Lupus is a very common disease
c) Lupus ist ansteckend	c) Lupus is contagious
d) Lupus sollte ausschließlich vom Hausarzt behandelt werden	d) Lupus should only be treated by a general practitioner
e) Lupus ist schlecht behandelbar	e) Lupus is difficult to treat
**2. Welcher der unten genannten Faktoren kann am ehesten einen Lupus-Schub auslösen?**	**2. Which of the following factors is most likely to trigger a lupus flare?**
a) Knochenbrüche	a) Bone fractures
b) Infektionen	b) Infections
c) Glutenhaltige Speisen	c) Foods containing gluten
d) Genuss von Cannabispräparaten (Marihuana)	d) Use of cannabis products (marijuana)
e) Kontakt mit Haustieren	e) Contact with pets
**3. In welchem Organsystem liegt die Ursache für Lupus?**	**3. In which organ system does lupus originate?**
a) Herz-Kreislauf	a) Cardiovascular system
b) Haut	b) Skin
c) Immunsystem	c) Immune system
d) Gelenke	d) Joints
e) Magen-Darm	e) Gastrointestinal tract
**4. Eine Nebenwirkung von Hydroxychloroquin betrifft welches Organ?**	**4. A side effect of hydroxychloroquine affects which organ?**
a) Niere	a) Kidney

b) Haut	b) Skin
c) Leber	c) Liver
d) Herz	d) Heart
e) Auge	e) Eye
**5. Welche Aussage zum Lupus stimmt?**	**5. Which statement about lupus is correct?**
a) Es gibt nur eine Ursache für Lupus	a) There is only one cause of lupus
b) Lupus ist heilbar	b) Lupus is curable
c) Der Lupus kann leicht mit anderen Erkrankungen verwechselt werden	c) Lupus can easily be mistaken for other diseases
d) Lupus bedeutet auf Lateinisch Hirsch	d) Lupus means 'deer' in Latin
e) Lupus betrifft vor allem Männer	e) Lupus mainly affects men
**6. Welches Merkmal des Lupus stellt für die Patienten eine große Gefahr dar und macht häufig eine intensive Behandlung notwendig?**	**6. Which feature of lupus poses a major risk to patients and often requires intensive treatment?**
a) Lähmungserscheinungen	a) Paralysis
b) Entzündung der Magenschleimhaut	b) Inflammation of the stomach lining
c) Schmetterlingserythem	c) Butterfly rash
d) Nierenentzündung	d) Kidney inflammation
e) Muskelschmerzen	e) Muscle pain
**7. Die Erhöhung welches Antikörpers kündigt am ehesten einen Schub an?**	**7. An increase in which antibody most likely indicates an upcoming flare?**
a) anti-CCP-Antikörper	a) Anti-CCP antibodies
b) anti-Scl-70-Antikörper	b) Anti-Scl-70 antibodies
c) anti-ds-DNA-Antikörper	c) Anti-dsDNA antibodies
d) anti-Gliadin-Antikörper	d) Anti-gliadin antibodies
e) anti-Zentromer-Antikörper	e) Anti-centromere antibodies

8. Welches der folgenden Krankheitszeichen tritt häufig beim Lupus auf?

a) Erhöhter Blutzucker

b) Schwerhörigkeit

c) Durchfall

d) Gallenkoliken

e) Gelenkentzündung

8. Which of the following symptoms commonly occurs in lupus?

a) Elevated blood sugar

b) Hearing loss

c) Diarrhea

d) Biliary colic

e) Joint inflammation

9. In welchem Alter tritt Lupus am häufigsten auf?

a) 0–20

b) 20–50

c) 50–80

d) über 80

e) In allen Lebensaltern gleich häufig

9. At what age does lupus most commonly occur?

a) 0–20

b) 20–50

c) 50–80

d) Over 80

e) Equally common at all ages

10. Welche Aussage zum sogenannten „Schmetterlingserythem“ stimmt?

a) Es kann durch Sonnenlicht entstehen

b) Es führt zu Haarausfall

c) Es ist eine Entzündung im Gelenk

d) Es tritt nur bei Männern auf

e) Es befällt besonders die Füße

10. Which statement about the so-called 'butterfly rash' is correct?

a) It can be triggered by sunlight

b) It leads to hair loss

c) It is an inflammation of a joint

d) It occurs only in men

e) It mainly affects the feet

11. Welche Aussage zu den Begleiterscheinungen von Lupus stimmt?

a) Gefäßverkalkung ist eine seltene Begleiterkrankung

b) Sport ist nicht zu empfehlen

11. Which statement about comorbidities of lupus is correct?

a) Atherosclerosis is a rare comorbidity

b) Exercise is not recommended

c) Impfungen mit Lebendimpfstoff werden empfohlen

c) Vaccination with live vaccines is recommended

d) Cortison fördert den Knochenabbau

d) Cortisone promotes bone loss

e) Sonnenlicht hat keinen Einfluss

e) Sunlight has no effect

12. Was kann die Krankheitsaktivität des Lupus verschlechtern?

12. What can worsen lupus disease activity?

a) Rauchen

a) Smoking

b) Sehr fettreiche Speisen

b) Very fatty foods

c) Ballaststoffreiche Kost

c) High-fiber diet

d) Ausdauertraining

d) Endurance training

e) Dieselabgase

e) Diesel exhaust

13. Welches Medikament gilt bei Lupus als gut verträglich und wird sehr häufig verschrieben?

13. Which medication is considered well tolerated and is frequently prescribed for lupus?

a) Betablocker

a) Beta blockers

b) Antibiotika

b) Antibiotics

c) Antikörper gegen TNF-alpha

c) Anti-TNF-alpha antibodies

d) Schmerzmittel wie Morphinpräparate

d) Painkillers such as morphine preparations

e) Azathioprin

e) Azathioprine

14. Welche Aussage zum Verlauf und zur Kontrolle von Lupus stimmt?

14. Which statement about the course and monitoring of lupus is correct?

a) Blutbild, Urinstatus und Autoantikörper sollten regelmäßig kontrolliert werden

a) Blood tests, urine tests, and autoantibodies should be checked regularly

b) Multivitaminpräparate sollten regelmäßig eingenommen werden

b) Patients should regularly take multivitamin supplements

c) Regelmäßige MRT-Untersuchungen der Nieren sind erforderlich

c) Regular MRI scans of the kidneys are required

d) Kontrolle maximal einmal jährlich

d) Monitoring should occur at most once per year

e) Medikamente nur im akuten Schub einnehmen	e) Medication should only be taken during acute flares
**15. Welche Aussage zur Schwangerschaft bei Lupus-Patienten stimmt?**	**15. Which statement about pregnancy in lupus patients is correct?**
a) Sehr häufig Zwillingsgeburten	a) Twin births are very common
b) Meist Kaiserschnitt	b) Delivery is almost always by cesarean section
c) Viele sind zeugungsunfähig	c) Many patients are infertile
d) Hydroxychloroquin kann während der Schwangerschaft eingenommen werden	d) Hydroxychloroquine can be taken during pregnancy
e) Schwangerschaft strikt vermeiden	e) Pregnancy should be strictly avoided
**16. Welche Veränderung zeigt sich typisch im Blutbild bei hoher Krankheitsaktivität?**	**16. Which change is typically seen in blood tests during high disease activity?**
a) Erhöhung des Cholesterins	a) Increased cholesterol
b) Erhöhung der Blutsenkungsgeschwindigkeit	b) Increased erythrocyte sedimentation rate
c) Erhöhung der Blutplättchen	c) Increased platelets
d) Erhöhung der roten Blutkörperchen	d) Increased red blood cells
e) Erhöhung der weißen Blutkörperchen	e) Increased white blood cells
**17. Welche Tagesdosis Cortison sollte langfristig angestrebt werden?**	**17. What daily dose of cortisone should be aimed for long-term?**
a) höchstens 5 mg	a) Maximum 5 mg
b) 7,5–20 mg	b) 7.5–20 mg
c) 20–50 mg	c) 20–50 mg
d) 50–100 mg	d) 50–100 mg
e) 100–200 mg	e) 100–200 mg

18. Welches Merkmal des Lupus der Mutter kann bei einer Schwangerschaft eine Gefahr für das Kind darstellen?

a) Raynaud-Syndrom

b) Hautbeteiligung

c) Antiphospholipid-Syndrom

d) Gelenkentzündung

e) Rippenfellentzündung

18. Which maternal lupus feature can pose a risk to the child during pregnancy?

a) Raynaud's syndrome

b) Skin involvement

c) Antiphospholipid syndrome

d) Joint inflammation

e) Pleurisy

19. Welcher Befund weist auf eine akute Nierenentzündung hin? Im Urin finden sich vermehrt:

a) Eiweiß (Protein)

b) Harnsäure

c) Bakterien

d) Glucose

e) Natrium und Kalium

19. Which finding indicates acute kidney inflammation (lupus nephritis)? Increased levels in urine of:

a) Protein

b) Uric acid

c) Bacteria

d) Glucose

e) Sodium and potassium

20. Welche Aussage zur Behandlung des Lupus ist richtig? Die Gabe von Cyclophosphamid…

a) wird vor allem in der Schwangerschaft empfohlen

b) führt zu einer Zunahme der weißen Blutkörperchen

c) ist schweren Krankheitsschüben vorbehalten

d) kann zu einer Medikamentensucht führen

e) kann zu vermehrtem Haarwachstum führen

20. Which statement about lupus treatment is correct? The use of cyclophosphamide…

a) is mainly recommended during pregnancy

b) leads to an increase in white blood cells

c) is reserved for severe disease flares

d) can lead to drug addiction

e) can cause increased hair growth
