# Supplementary material for: Increasing Disease-Specific Knowledge in Patients with SLE Through a Structured One-Day Seminar: Results of a Randomized, Controlled Study
Source: Healthcare (Basel). 2026 Apr 30;14(9):1209. doi: 10.3390/healthcare14091209 (PMC13163604; doi:10.3390/healthcare14091209)
Supplement: Supplementary file 1 [file healthcare-14-01209-s001.zip › Table S1.pdf]

Table S1

Health-related lifestyle behavior at baseline and three months after the educational seminar. Data is given as absolute number and relative percentage. An average score is given for the AHEI (alternative healthy eating index). The maximum possible score is 9.

		Baseline	Follow-up (3 months)
**Sun protection**	never	0 (0 %)	0 (0 %)
	seldom	0 (0 %)	2 (5 %)
	most of the time	25 (66 %)	20 (49 %)
	always	13 (31 %)	16 (39 %)
	no answer	1 (3 %)	1 (3 %)
**Physical activity**	never	6 (15 %)	7 (18 %)
	< 1h	8 (21%)	8 (21 %)
	1 to 2 hours	12 (31 %)	14 (36 %)
	2 to 4 hours	6 (15 %)	6 (15 %)
	> 4 hours	6 (15 %)	4 (10 %)
	no answer	1 (3 %)	0 (0 %)
**AHEI**	average score	5.07 (95%-CI 4.1-6.0)	5.22 (95%-CI 4.4-6.1)
**Smoking**	never	18 (46 %)	18 (46 %)
	ex-smoker	13 (33 %)	13 (33 %)
	smoker	8 (21 %)	8 (21 %)
