# Supplementary figures and images for: Increasing Disease-Specific Knowledge in Patients with SLE Through a Structured One-Day Seminar: Results of a Randomized, Controlled Study
Source: Healthcare (Basel). 2026 Apr 30;14(9):1209. doi: 10.3390/healthcare14091209 (PMC13163604; doi:10.3390/healthcare14091209)

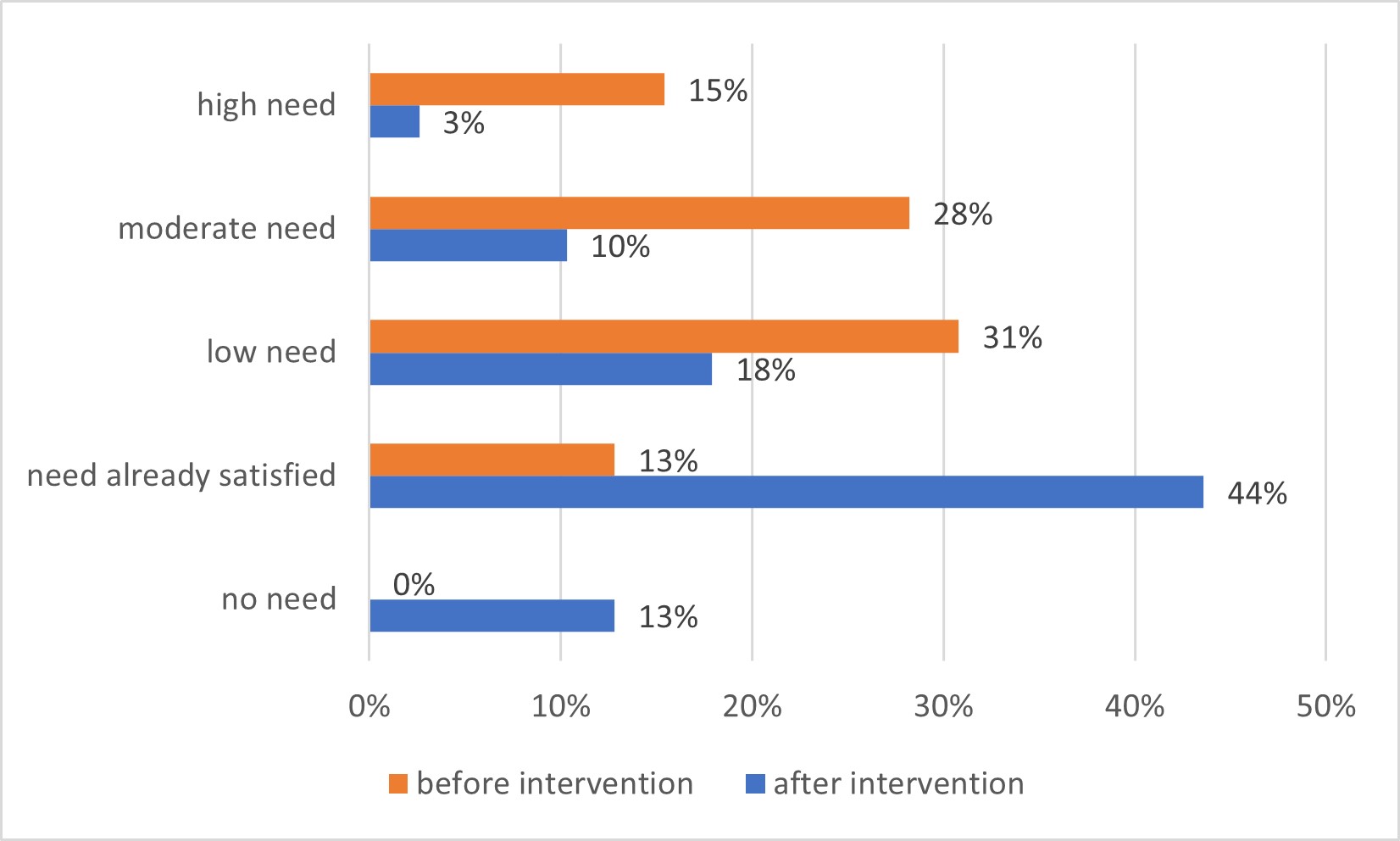

Supplement: Supplementary file 1 [file healthcare-14-01209-s001.zip › Figure S1 Need for health information.jpg]

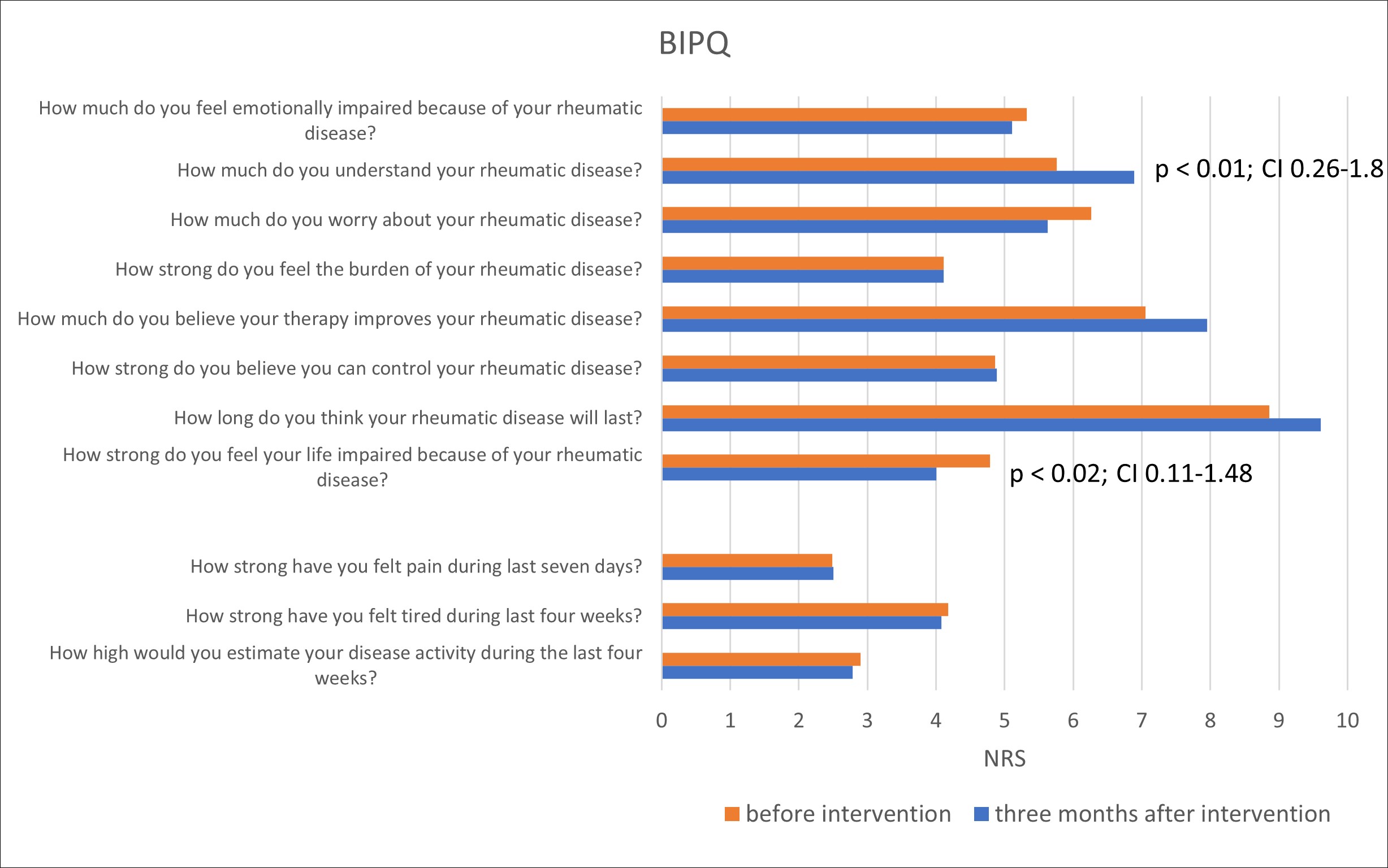

Supplement: Supplementary file 1 [file healthcare-14-01209-s001.zip › Figure S2 Illness perception.jpg]

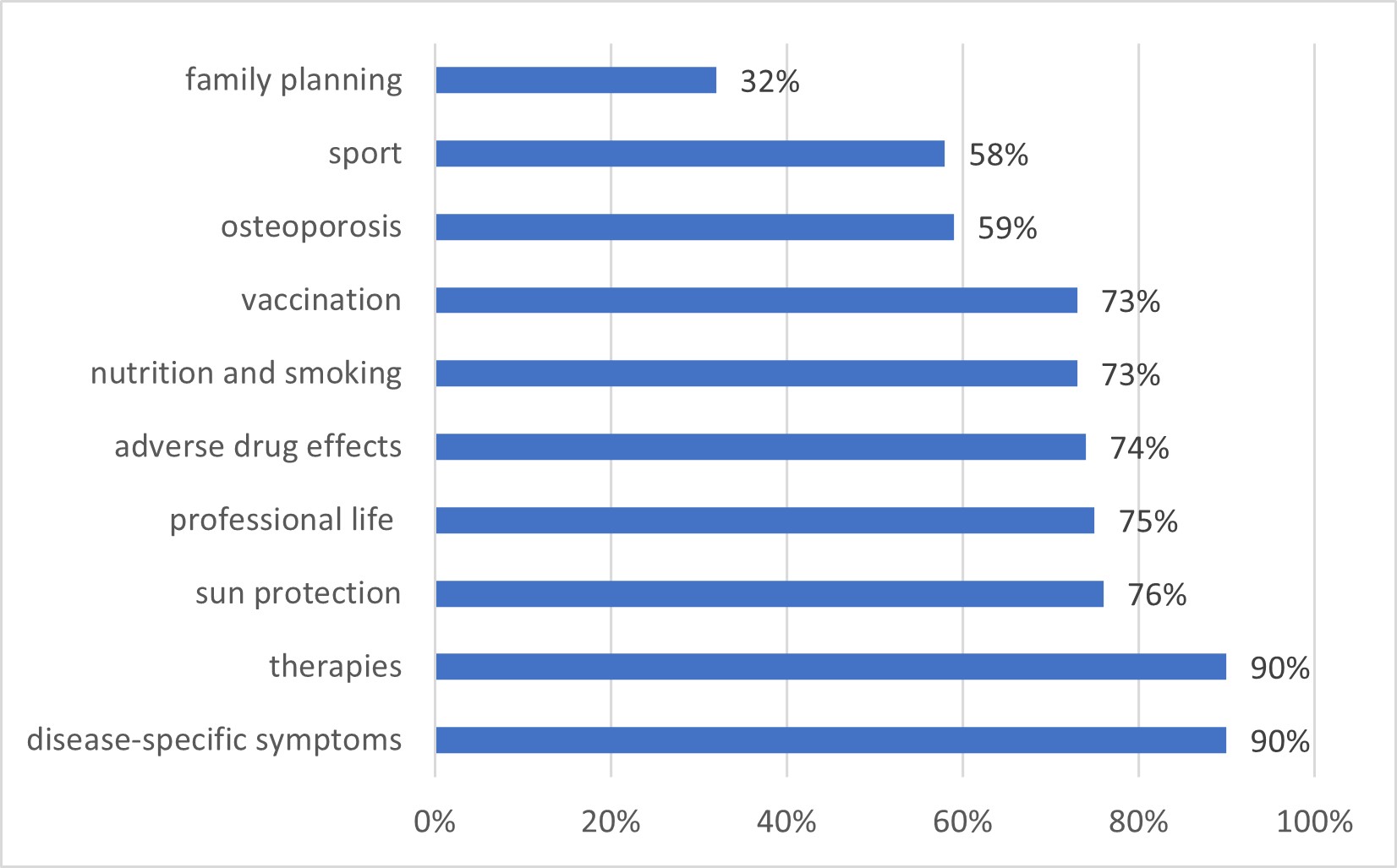

Supplement: Supplementary file 1 [file healthcare-14-01209-s001.zip › Figure S3 Important topics.jpg]

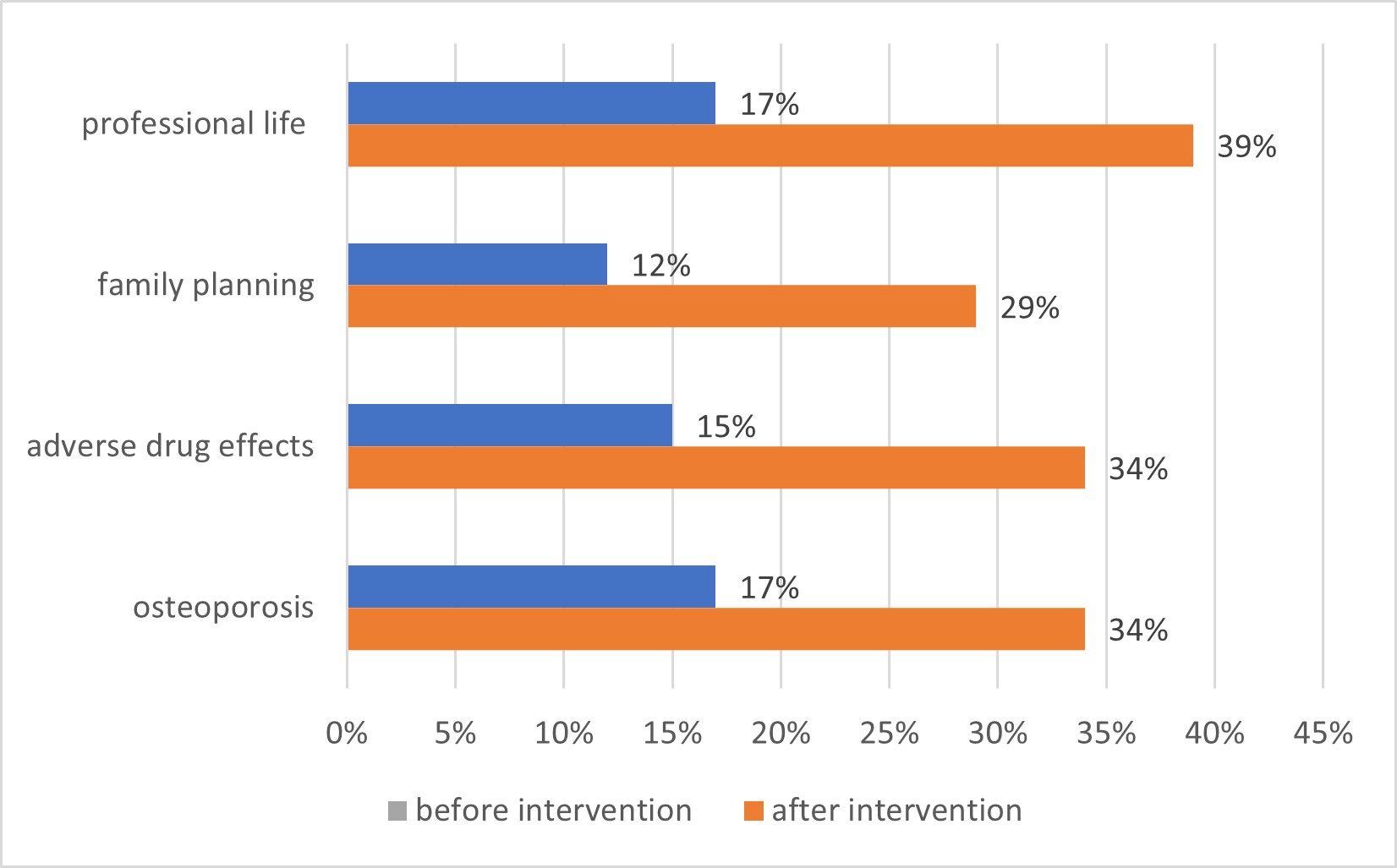

Supplement: Supplementary file 1 [file healthcare-14-01209-s001.zip › Figure S4 Information content.jpg]

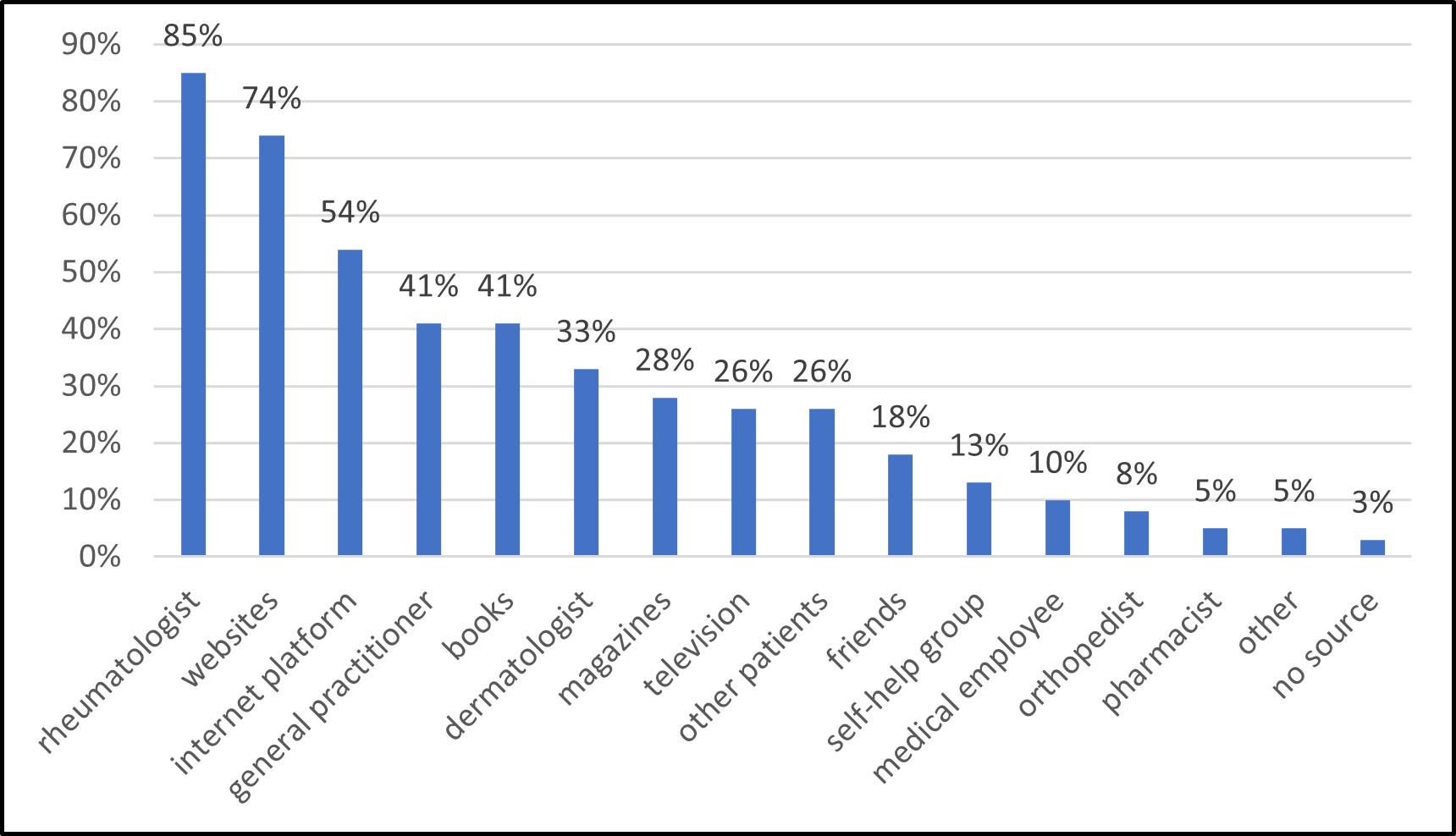

Supplement: Supplementary file 1 [file healthcare-14-01209-s001.zip › Figure S5 Sources of disease-related information.jpg]
